# Supplementary material for: Unraveling the Polysaccharide Biosynthesis Potential of Ganoderma lucidum: A Chromosome-Level Assembly Using Hi-C Sequencing
Source: J Fungi (Basel). 2023 Oct 16;9(10):1020. doi: 10.3390/jof9101020 (PMC10608111; doi:10.3390/jof9101020)
Supplement: Supplementary file 1 [file jof-09-01020-s001.zip › Supplementary materials.pdf]

# Supplementary materials

**Files** 1
**Notes** 2
Testing the scheme for identifying the genes putatively involved in polysaccharide biosynthesis using a published Ganoderma genome 2
**Tables** 3
**Figures** 5
Supplementary references 6

Files

Supplementary File 1 - List of 44 genes functionally associated with the synthesis of polysaccharides, annotated using GO terms.
Supplementary File 2 - Dictionary of relevant GO terms associated with the synthesis of polysaccharides mentioned in Supplementary File 1. The dictionary was compiled by identifying genes associated with polysaccharide metabolism in the annotated reference genome based on the KEGG record "Metabolism of Complex Carbohydrates." It includes an aggregation of GO terms corresponding to these genes. The reference KEGG annotation was utilized for more accurate annotation instead of the predicted GO annotation when searching for homologous candidate genes.
Supplementary File 3 - Amino acid sequences of transcripts from genes involved in the synthesis of polysaccharides, identified through the search for homologous candidate genes using the reference KEGG annotation.
Supplementary File 4 - The .hic file containing the contact map of the *G. lucidum* genome after manual reordering.
Supplementary File 5 - The .asm file providing the contact map of the *G. lucidum* genome with labeling for each quasichromosome.
Supplementary File 6 - Table presenting the length and coordinates of each quasichromosome in the *G. lucidum* genome.
Supplementary File 7 - The secondary metabolite biosynthesis predictions according to antiSMASH (in Genbank format).

Notes

Testing the scheme for identifying the genes putatively involved in polysaccharide biosynthesis using a published *Ganoderma* genome

The genome of *Ganoderma* sp. 10597 was obtained from the following public source: https://genome.jgi.doe.gov/portal/Gansp10597SS1_FD/Gansp10597SS1_FD.info.html.

Initial attempts to identify relevant genes (transcripts encoding proteins similar to Och1p, Van1p, Anp1p, Mnn9p, Mnn2p, Mnn6p) through BLAST analysis of this genome did not yield significant results (all candidate similarities had e-values exceeding $10^{-3}$).

Considering the possibility that non-orthologous proteins might be responsible for the relevant functions in this organism, gene profiles based on gene ontology (GO) annotations were compiled for each gene, including the proteins of interest. Subsequently, a search was conducted in the genome for genes with transcripts exhibiting similar annotations.

A total of 27 genes were identified whose transcript profiles intersected with the profiles of the target proteins. After expert curation, the number of genes potentially involved in polysaccharide synthesis was reduced to 19 (refer to Table S1). Each transcript was then subjected to a BLAST search to find the closest known homolog, refining its function. All 19 proteins were found to have annotated homologs in the *nr* database. Furthermore, the Enzyme Commission number (EC) nomenclature was used to categorize each protein and further refine the potentially catalyzed reaction.

Analysis of the KEGG database based on the obtained EC numbers allowed for the identification of metabolic pathways similar to the ones of interest (see Figures S1 and S2). Additionally, through a comparison of protein sequences involved in similar reactions found in the literature (Onoue et al. 2018), homology was discovered between some of the identified proteins and the transcripts of the genes jgi|Gansp1|55798|e_gw1.11.587.1, jgi|Gansp1|56496|e_gw1.11.376.1, and jgi|Gansp1|129696|fgenesh1_pm.7_#_370, which are homologous to Afu5g12160 and Afu5g02740 proteins.

Tables

**Table S1 - Initially identified candidate genes potentially associated with the synthesis of the target polysaccharides.**

| Gene ID in Ganoderma sp. 10597 | BLAST related protein | EC | Type of enzyme |
|---|---|---|---|

| | | | |
|---|---|---|---|
| >jgi\|Gansp1\|38124\|e_gw1.1.2377.1 | Alpha-1,2-glucosyltransferase ALG10-A | 2.4.1.- | hexosyltransferases |
| >jgi\|Gansp1\|41514\|e_gw1.2.2688.1 | Dolichyl pyrophosphate Glc1Man9GlcNAc2 alpha-1,3-glucosyltransferase | 2.4.1.- | hexosyltransferases |
| >jgi\|Gansp1\|42603\|e_gw1.2.2497.1 | Dolichyl pyrophosphate Glc1Man9GlcNAc2 alpha-1,3-glucosyltransferase | 2.4.1.109 | Dolichyl-phosphate-mannose--protein mannosyltransferase. |
| >jgi\|Gansp1\|51293\|e_gw1.7.902.1 | Dolichyl-phosphate-mannose--protein mannosyltransferase 1 | 2.4.1.130 | Dolichyl-phosphate-mannose-glycolipid alpha-mannosyltransferase. |
| >jgi\|Gansp1\|51570\|e_gw1.7.313.1 | GPI mannosyltransferase 1 | 2.4.1.- | hexosyltransferases |
| >jgi\|Gansp1\|55798\|e_gw1.11.587.1 | Glycolipid 2-alpha-mannosyltransferase 2 | 2.4.1.131 | Glycolipid 2-alpha-mannosyltransferase. |
| >jgi\|Gansp1\|56496\|e_gw1.11.376.1 | alpha-1,2-mannosyltransferase | 2.4.1.131 | Glycolipid 2-alpha-mannosyltransferase. |
| >jgi\|Gansp1\|69736\|estExt_Genewise1.C_6_t10369 | oligosaccharyl transferase stt3 subunit | 2.4.1.119 | Dolichyl-diphosphooligosaccharide--protein glycosyltransferase. |
| >jgi\|Gansp1\|73716\|estExt_Genewise1.C_9_t20352 | GPI mannosyltransferase 4 | 2.4.1.- | hexosyltransferases |
| >jgi\|Gansp1\|84501\|estExt_Genewise1Plus.C_4_t10328 | GDP-Man:Man(3)GlcNAc(2)-PP-Dol alpha-1,2-mannosyltransferase | 2.4.1.- | hexosyltransferases |
| >jgi\|Gansp1\|88381\|estExt_Genewise1Plus.C_7_t10263 | Dolichyl-phosphate-mannose--protein mannosyltransferase 2 | 2.4.1.109 | Dolichyl-phosphate-mannose--protein mannosyltransferase. |
| >jgi\|Gansp1\|88625\|estExt_Gene | GPI mannosyltransferase 3 | 2.4.1.- | hexosyltransferases |

| | | | |
|---|---|---|---|
| wise1Plus.C_7_t20055 | | | |
| >jgi\|Gansp1\|127772\|fgenesh1_pm.4_#_322 | ALG6 ALG8 glycosyltransferase | 2.4.1.- | hexosyltransferases |
| >jgi\|Gansp1\|129696\|fgenesh1_pm.7_#_370 | Glycolipid 2-alpha-mannosyltransferase 1 | 2.4.1.131 | Glycolipid 2-alpha-mannosyltransferase. |
| >jgi\|Gansp1\|132299\|fgenesh1_pm.13_#_149 | Beta-1,4-mannosyl-glycoprotein 4-beta-N-acetylglucosaminyltransferase | 2.4.1.144 | Beta-1,4-mannosyl-glycoprotein 4-beta-N-acetylglucosaminyltransferase |
| >jgi\|Gansp1\|113974\|fgenesh1_kg.1_#_1092_#_isotig08803 | Alpha-1,2-mannosyltransferase ALG9 | 2.4.1.- | hexosyltransferases |
| >jgi\|Gansp1\|115010\|fgenesh1_kg.2_#_583_#_isotig10382 | Ribophorin I | 2.4.1.119 | Dolichyl-diphosphooligosaccharide--protein glycosyltransferase. |
| >jgi\|Gansp1\|118777\|fgenesh1_kg.6_#_527_#_isotig08567 | O-mannosyltransferase | 2.4.1.109 | Dolichyl-phosphate-mannose--protein mannosyltransferase. |
| >jgi\|Gansp1\|123003\|fgenesh1_kg.12_#_593_#_isotig07483 | UDP-glucose:glycoprotein glucosyltransferase | 2.4.1.- | hexosyltransferases |

Figures

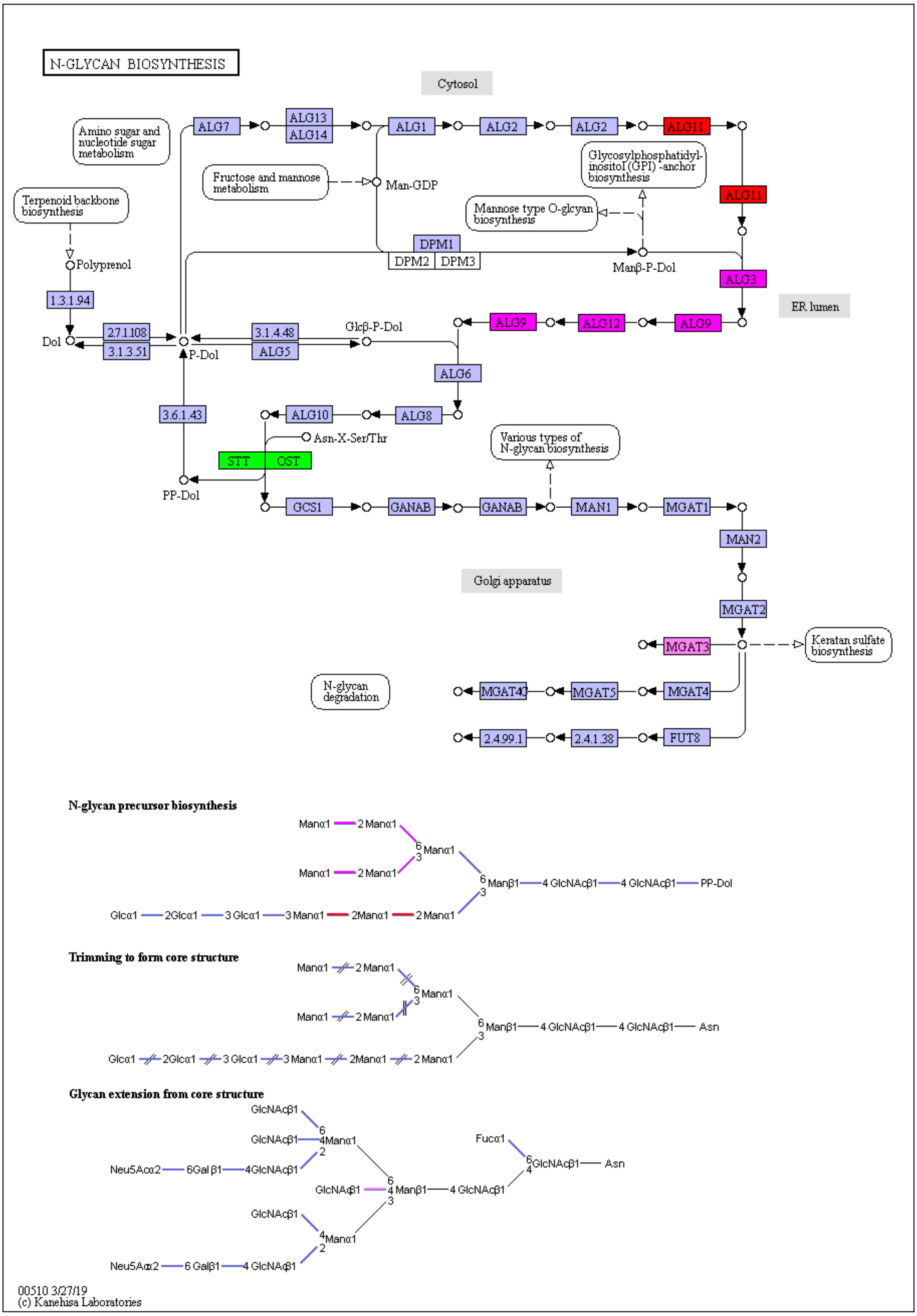


**Figure S1 - Identified genes shown on the "N-glycan biosynthesis" KEGG pathway.** The nodes corresponding to the identified ECs are highlighted: 2.4.99.18 - green; 2.4.1.131 - red; 2.4.1.144 - violet; 2.4.1.130, 2.4.1.258 - 2.4.1.261 - purple.

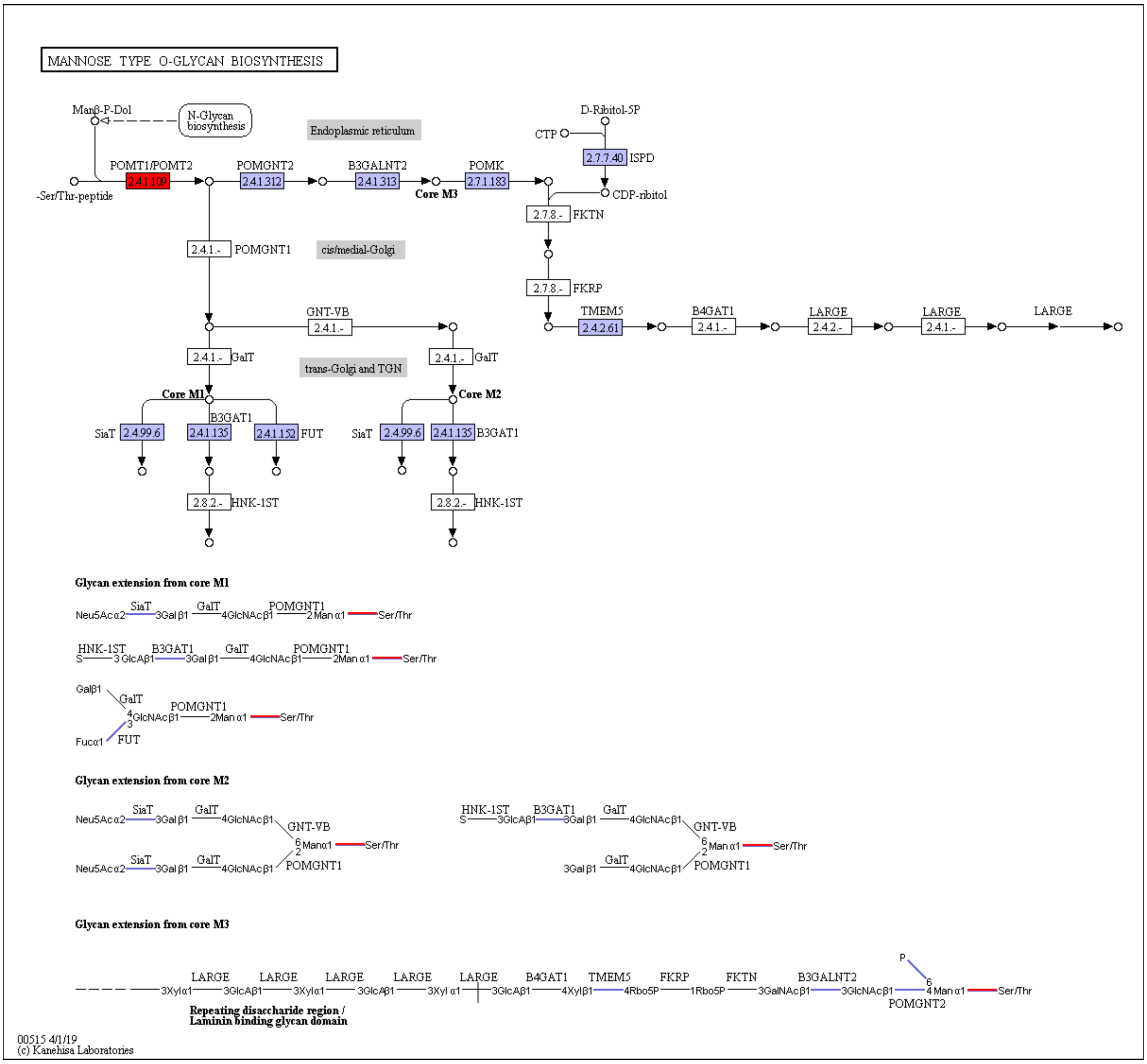


**Figure S2 - Identified genes shown on the "Mannose type O-glycan biosynthesis" KEGG pathway.** The sole identified gene - EC 2.4.1.109 - is highlighted in red.

Supplementary references

Onoue, Takuya, Yutaka Tanaka, Daisuke Hagiwara, Keisuke Ekino, Akira Watanabe, Kazuyoshi Ohta, Katsuhiko Kamei, Nobuyuki Shibata, Masatoshi Goto, and Takuji Oka. 2018. "Identification of Two Mannosyltransferases Contributing to Biosynthesis of the Fungal-Type Galactomannan $\alpha$-Core-Mannan Structure in Aspergillus Fumigatus." *Scientific Reports* 8 (1): 16918.
